# The Characteristics and Dynamics of Cyanobacteria–Heterotrophic Bacteria Between Two Estuarine Reservoirs – Tropical Versus Sub-Tropical Regions

**DOI:** 10.3389/fmicb.2018.02531

**Published:** 2018-11-06

**Authors:** Zheng Xu, Shu Harn Te, Yiliang He, Karina Yew-Hoong Gin

**Affiliations:** ^1^School of Environmental Science and Engineering, Shanghai Jiao Tong University, Shanghai, China; ^2^NUS Environmental Research Institute (NERI), National University of Singapore, Singapore, Singapore; ^3^Department of Civil and Environmental Engineering, National University of Singapore, Singapore, Singapore

**Keywords:** Illumina MiSeq sequencing, microbial community, estuarine reservoir, cyanobacteria–heterotrophic bacteria, molecular ecological network

## Abstract

In this study, Illumina MiSeq sequencing technique was employed to explore the characteristics and dynamics of cyanobacteria–heterotrophic bacteria between two estuarine reservoirs in sub-tropical (reservoir A in Shanghai) and tropical (reservoir B in Singapore) regions. The results indicated that significant differences in bacterial community composition were found between two estuarine reservoirs, which influenced by varied environmental variables. The environmental heterogeneity in reservoir A was much higher, which indicated that the composition of bacterial community in reservoir A was more complex. In contrast, reservoir B provided a suitable and temperate water environment conditions for bacterial growth, which resulted in higher community diversity and less co-exclusion correlations. The molecular ecological network indicated that the presence of dominant bacterial community in each of the reservoir were significant different. These differences mainly reflected the responses of bacterial community to the variations of environmental variables. Although *Synechococcus* was the dominant cyanobacterial species in both reservoirs, it exhibited co-occurrence patterns with different heterotrophic bacteria between reservoirs. In addition, the cyanobacteria–heterotrophic bacteria interaction exhibited highly dynamic variations, which was affected by nutrition and survive space. Also, the co-occurrence of *Microcystis* and *Pseudanabaena* found in reservoir B implied that the non-N-fixing *Microcystis* accompanied with N-fixing *Pseudanabeana* occurrence in freshwater lakes, so as to better meet the demand for nitrogen source.

## Introduction

In aquatic ecosystems, the balanced relationships of cyanobacteria–heterotrophic bacteria play important roles in maintaining aquatic ecologic stability (Cole, 1982). However, the unbalanced relationships of cyanobacteria–heterotrophic bacteria could further lead to harmful cyanobacterial blooms. In aquatic ecosystems, the variations of cyanobacteria–heterotrophicbacteria compositions were closely correlated with the whole bacterial community changes, which was strongly affected by external water environmental conditions. Previous studies also proved that the changes of environmental conditions could directly or indirectly affect the relationships of cyanobacteria–heterotrophic bacteria in aquatic ecosystems, likewise, the dynamics of cyanobacteria–heterotrophic bacteria could also strongly reflect the water quality status (Celussi and Cataletto, 2007; Newton et al., 2007; Wu et al., 2007; Xu et al., 2018).

Estuaries are important water sources for numerous cities such as Shanghai, Hong Kong, and Singapore, which located in estuarine areas around the world (Shu and Gin, 2011; Yue and Tang, 2011; Sun et al., 2014). Due to the special geographical location of estuarine areas, the estuary ecosystems are simultaneously affected by terrestrial and marine ecosystems (Pritchard, 1967). Thus, the estuarine ecosystems have higher complex structure and diversity than other aquatic ecosystems including freshwater lakes and rivers (Vasconcelos et al., 2015). These complex environmental conditions significantly affect the bacterial community composition in these areas (Dang et al., 2008; Dang and Lovell, 2016). Especially, changes of bacterial community composition are expected to cause functional consequences, which can result in substantial alteration of ecosystems, such as decreases in primary productivity or changes of nitrogen (N) cycle (Dang et al., 2010, 2013). More important, these changes may affect the correlations of cyanobacteria–heterotrophic bacteria, which might cause potential harmful cyanobacteria blooms.

In our study, Shanghai and Singapore, as metropolitan cities located in estuary areas with different geographical characteristics, were chosen as our study sites. High-throughput sequencing (HTS) techniques were used to evaluate the diversity and variations of bacterial community composition in estuaries between different ecological regions. Especially, the molecular ecological network was implemented to explore the composing characteristics of cyanobacteria–heterotrophic bacteria between these estuarine reservoirs. We hope this research could promote the understanding of interactions within cyanobacteria–heterotrophic bacteria and how environmental factors affect their compositions in estuary reservoirs.

## Materials and Methods

### Site Description and Sample Collection

Two typical estuarine reservoirs in Shanghai (reservoir A) and Singapore (reservoir B) were selected in our studies, which represented estuaries ecosystems in sub-tropical and tropical regions, respectively (Supplementary Figure S1). Reservoir A (31°28′N, 121°36′E) is the largest estuarine reservoir in China in the Yangtze estuary with a total area of almost 66 km^2^ and a depth ranging from 2.5 to 13.5 m (Ou et al., 2013). It was constructed in 2011 for alleviating the drinking water shortage in Shanghai city (Liu et al., 2009; Jin et al., 2013a,b). Reservoir B (1°17′N, 103°51′E) is the largest multifunctional estuarine reservoir in Singapore completed in 2008 providing recreation, flood control and water storage for the country (Teng, 2009). This reservoir is located in the heart of the city and surrounded by commercial, residential, and industrial areas (Glover and Onn, 2008). The catchment area of this reservoir is about 100 km^2^, and the water body surface area is 2.4 km^2^ (Te et al., 2017).

Two sampling sites in each reservoir were selected to represent different hydrological conditions: QI and QE sites represented the midstream and downstream location of reservoir A, respectively. While in reservoir B, MA and MB represented the meeting points of different tributaries and main reservoir. All water samples were collected monthly for a year (May 2014 to April 2015) at a depth of 0.5 m below the surface water from these four sites.

### Water Quality and Environmental Factors

Water temperature and pH were measured *in situ* using a YSI 6600 V2 multi-parameter water quality analyzer (YSI Inc.). Equal volumes of water (5 L) were collected from each site and stored in brown bottles previously cleaned by 10% nitric acid solution. All water samples were stored on ice (∼4°C) before further analysis.

Water quality parameters including turbidity (NTU), chlorophyll-a, total phosphorous (TP) and total nitrogen (TN) were analyzed immediately according to water and wastewater monitoring analysis methods (Wei, 2002), upon arrival at the labs in both China and Singapore. The concentrations of positive ions including Ca^2+^ and Mg^2+^ were detected using Inductively Coupled Plasma (ICP) spectroscopy. The concentrations of negative ions including chloride and sulfate were determined by Metrohm 830 Ion Chromatography (Jin et al., 2013b). In addition, the total rainfall 1 day (rain 1 d), 5 days (rain 5 d), and 30 days (rain 30 d) prior to the sampling dates were obtained from National Environmental Agency (NEA) website in Singapore and Meteorological Data Service Center (CMDC) website in China.

### Molecular Analysis

The genomic DNA was extracted from 500 mL of water sample from each site by using E.Z.N.A. Water DNA Kit (Omega, Inc.) according to the manufacturer’s specifications. The extracted DNA was detected using Qubit 3.0 fluorometer (Invitrogen, Inc.) and frozen at -20C for further analysis. The PCR amplification was performed according to the two-step target amplicon sequencing protocol (Green et al., 2015). First, the primers, 926wF and 1392R (Rinke et al., 2014) combined with common sequence tags CS1 and CS2 at the 5′ position, were used to amplify the 16S rRNA gene V6-8 region. The PCR reaction mixtures containing 9 μL of AccuPrime Pfx SuperMix (Invitrogen, Inc.), 400 nM of each primer (forward/reverse) and 1 μL DNA template. The PCR cycle included an initial denaturation at 95°C for 5 min, followed by 28 cycles of denaturation at 95°C for 15 s, annealing at 60°C for 30 s, extension at 68°C for 40 s, and a final elongation at 68°C for 5 min. After PCR amplification, all PCR products were sent to the University of Illinois, Resources Center-DNA Services Facility for further processing. During the second stage, additional PCR amplification including eight cycles was implemented to combine sequencing adaptor and barcodes. After that, paired-end amplicon sequencing (2 × 250 bp) was sequenced on Illumina MiSeq platform by using the specific primers, CS1_926wF and CS2_1392R.

Raw sequencing data (NCBI BioProject: PRJNA397362) were processed according to the MiSeq standard operating procedure by using Mothur (version 1.35.1^1^). During this stage, the length of sequences which were too long or too short, and sequences containing Ns or any ambiguous bases pairs were eliminated. Remaining sequence reads were aligned against the SILVA database (Release128^2^) and pre-clustered at 2%. UCHIME algorithm was implemented to identify and remove chimeric sequences. Remaining reads were assigned and taxonomically classified by using SILVA database v128. Sequences passing the quality control steps were clustered to operational taxonomic units (OTUs) based on the average neighbor algorithm at 3% dissimilarity. The taxonomy of each OTU was assigned through generating a consensus based on the taxonomic identity of these OTUs within each group. Following these sequencing steps, subsampling was performed to ensure consistency across the whole data set.

### Quantitative Analysis of Bacteria and Cyanobacteria Abundance

The quantification of cyanobacterial 16S rRNA gene was carried out in a StepOnePlus real-time PCR (qPCR) system using primers and probes designed in a previous study (Te et al., 2017). The reaction mix consisted of 10 μL of FastStart Universal Probe Master (Roche), 0.8 μM of each forward/reverse primer, 0.2 μM of Taqman probe (FAM) and 2 μL of DNA template in a total reaction of 20 μL. The profile included an enzyme activation at 95°C for 10 min and 40 cycles of 15 s at 95°C, 25 s at 56°C, and 25 s at 72°C. The quantification standard curve was established using a 10-fold serially diluted plasmid (Integrated DNA Technologies) cloned with the target. The GCNs were calculated from the external standard curve established on plasmid clones with linear quantification range between 30 and 3 × 10^8^ copies of gene per reaction.

### Statistical Analyses

Before statistical analysis, both biological and environmental data were first preprocessed, and standardized. The relative abundance of bacterial OTUs were square-root-transformed and environmental data were normalized. The similarity matrices of biological data and environmental characteristics were then calculated based on Bray–Curtis similarity and Euclidean distance, respectively. The contribution of each measured environmental variable to the variation within the bacterial community composition in each reservoir were evaluated based on distance-based linear model (DistLM) implemented in PRIMER v7 (PRIMER-E Ltd., Unite Kingdom) (Clarke, 1993; Clarke and Gorley, 2006). To detect spatial and temporal differences of bacterial community between different samples, permutation multivariate analysis of variance (PERMANOVA) was performed PRIMER v7. RELATE-BEST were used to evaluate the degrees of correlation between environmental variables and bacterial OTUs. In addition, the species richness and inverse Simpson index of each sample was also calculated from normalized OTUs using PRIMER v7. To further investigate the correlations between distributions of samples and environmental variables in each reservoir, non-metric multidimensional scaling (nMDS) was conducted by calculating the Spearman correlation coefficient based on the Bray–Curtis similarity.

In each reservoir, the bacterial OTUs which were observed in at least five samples (>20%) and contributed at least 1% to the sample, were selected from the raw OTU data set. The observed abundance of these bacterial OTUs was not altered within any sample. The selected OTUs were further used for assessing the degree of association with respect to environmental parameters across the entire sampling period based on Pearson’s correlation coefficient (*r*). Both Pearson’s correlation coefficient (*r*) and *P*-value were calculated pair-wise for each correlation based on rcor.test algorithm from ltm package as implemented in R v 3.3.2 (Woodhouse et al., 2016). The false discovery of *P*-value was kept below 5% based on the Benjamini–Hochberg procedure during our research (Benjamini and Hochberg, 1995). The obtained correlations were used to form a visualized network by using Gephi package (version 0.9.2^3^). A circular layout network was constructed based on Fruchterman Reingold algorithm, further implemented on modularity calculation and classification for each node inside the network. During the network, the betweenness centrality and other metric values of nodes and edges were also calculated by using network analysis plug-in (Bastian et al., 2009). In the network, nodes represented different bacterial OTUs and environmental factors. Edges represented the correlations between different nodes. Betweenness centrality was used to measure the centrality of nodes based on the shortest paths. Compared with other topological parameters, betweenness centrality was an important indicator to measure the role of node within the network (Yu et al., 2007). Nodes with high betweenness centrality (large nodes) show high centrality—i.e., higher control over the network. Thus, we selected betweenness centrality as measure indicator to evaluate the contributions of different nodes (including bacterial OTUs and environmental factors) in the network.

## Results

### Water Quality and Environmental Parameters in the Estuarine Reservoirs A and B

During the sampling period, seasonal variations in reservoir A was observed with higher temperatures recorded from May to October and lower temperature from November to April (Supplementary Figure S2A). The highest temperature (28.8°C) was found in July at site QI and the lowest (6.6°C) in February at site QE. Besides, the average of pH in reservoir A was 8.4 across the whole year, and the highest value of pH (9.3) occurred in June at QI site, the lowest value of pH (7.8) occurred in October at QE site (Supplementary Figure S2B). The concentration of chlorophyll-a ranged from 4.15 μg/L (May at site QE) to 48.52 μg/L (August at site QE) in reservoir A, and exhibited relative higher concentration from July to August (Supplementary Figure S2C). In contrast, reservoir B showed minor fluctuation in temperature ranging from 27.3 to 32.2°C throughout the whole year and no significant trend in pH was observed. The chlorophyll-a concentration, were much higher from May to November with maximum (52.3 μg/L) in October at site MB, and decreased from December to April with lowest values (9.4 μg/L) observed in January. The cyanobacterial 16S rRNA gene copies were maintained at relatively higher level in reservoir A (∼8.5 × 10^4^ gene copies) than in reservoir B (∼3.7 × 10^4^ gene copies) except in November (Supplementary Figure S2D). In addition, reservoir A exhibited much higher TN concentrations (0.93–2.55 mg/L) compared to reservoir B (0.59–1.2 mg/L) (Supplementary Figure S2E). These differences were more significant in the winter than summer period. The TP concentrations were slightly higher in reservoir B (0.024–0.108 mg/L) than in reservoir A (0.00525–0.0837 mg/L), except in July and August at QI site in reservoir A (Supplementary Figure S2F). Based on the variations of TN and TP concentrations, the average of TN/TP ratios in reservoir A were maintained between 41.1 and 71.6 during May to October, but increased rapidly from November to April to 150–155 (Supplementary Figure S2G). In contrast, the trend in TN/TP ratio was relatively stable and lower in value for reservoir B, with an average of 18.69. The turbidity in reservoir A (12.1 NTU) was also significantly higher than in reservoir B (6.3 NTU) from May to November during the growing season in Shanghai, but the turbidity increased rapidly and was kept at higher level from December to April in reservoir B (18.6 NTU at site MA) (Supplementary Figure S2H). Changes in ion concentrations (Ca^2+^, Mg^2+^, chloride and sulfate) were different for these two reservoirs but these ions were well-correlated to each other (Supplementary Figures S2I–L). Ca^2+^, Mg^2+^ and sulfate were much higher in reservoir A, especially in May, December to April (Supplementary Figures S2I,J,L). In contrast, the concentration of chloride was maintained at higher level in reservoir B than reservoir A (Supplementary Figure S2K). The data of other environmental factors can be found in supplementary materials (Supplementary Tables S1, S2).

### Microbial Community Structure

**FIGURE 1:**
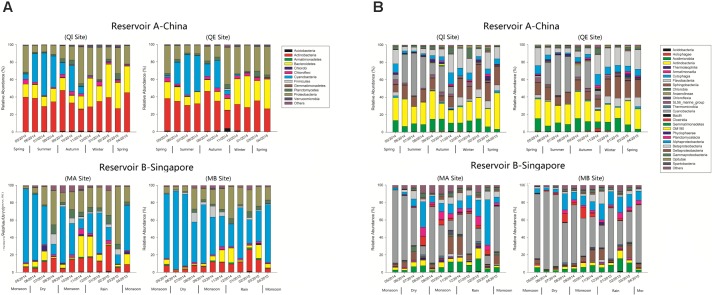
**(A,B)** Relative abundance of 16S rRNA bacterial OTUs in reservoir A (China) and B (Singapore) across the whole sampling period (**A**: Phylum level, **B**: Class level).

After quality control, a total of 2,900,208 high quality sequences were observed both from reservoir A and B, with an average of 60,421 sequences in each sample. All these sequences were aligned against the SILVA database, and finally total of 2,093 bacterial OTUs were identified. These bacterial OTUs were belonged to 30 bacterial phyla and 249 genera. Among these bacterial OTUs, 20 bacterial phyla and 127 genera were observed in reservoir A. In contrast, 29 bacterial phyla and 198 genera were found in reservoir B. In reservoir A, the most dominant bacterial phylum was Actinobacteria (33.6%), and followed by Proteobacteria (22.7%), Bacteroidetes (20.0%), and Cyanobacteria (15.0%) (Figure 1A). But in reservoir B, the most dominant bacteria phylum was Cyanobacteria (47.0%), followed by Proteobacteria (20.7%), Actinobacteria (10.8%), and Bacteroidetes (6.9%).

During the further classification levels, most of actinobacterial OTUs were classified as Actinobacteria (class level) in reservoir A, but more actinobacterial OTUs were classified as Acidimicrobiia in reservoir B (Figure 1B). In reservoir A, most Bacteroidetes were composed by Sphingobacteriia and Flavobacteriia. Whereas, Bacteroidetes were most classified as Sphingobacteriia in reservoir B. In addition, the β-Proteobacteria exhibited higher relative abundance in reservoir A, but the α-Proteobacteria and δ-Proteobacteria revealed higher relative abundance in reservoir B.

**FIGURE 2:**
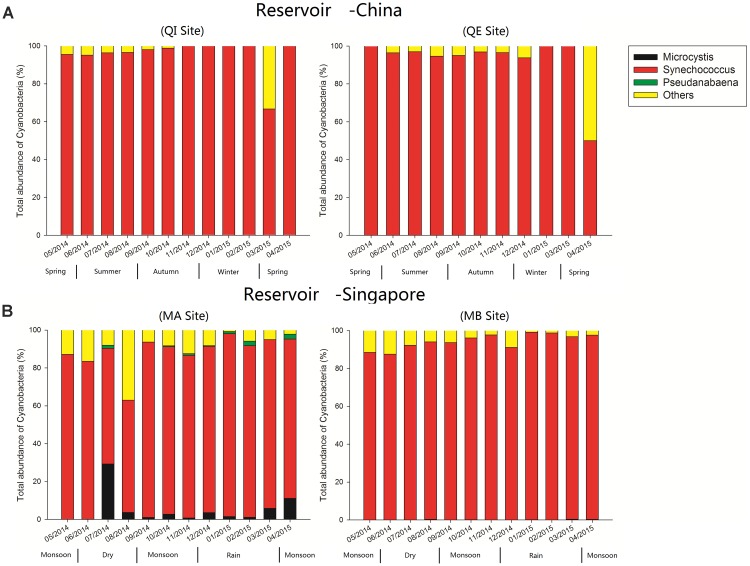
Relative abundance of different cyanobacterial genera over time in reservoir **A** (China) and **B** (Singapore).

The results of high-throughput sequence indicated that the *Synechococcus* was the most dominant cyanobacterial genus, and the relative abundance of *Synechococcus* accounting for an average of 94% of the total relative abundance of all cyanobacterial OTUs in reservoir A (Figure 2). For reservoir B, the *Synechococcus* also emerged as the dominant cyanobacterial genus, and the relative abundance of *Synechococcus* accounting for almost 89% of total relative abundance of all cyanobacterial OTUs (Figure 2). In addition, the *Microcystis* was detected with higher relative abundance at site MA in July (∼29.3%) and April (∼11.1%). Other genera such as *Pseudanabaena*, was also detected in reservoir B but the relative abundances less than 3%.

**FIGURE 3:**
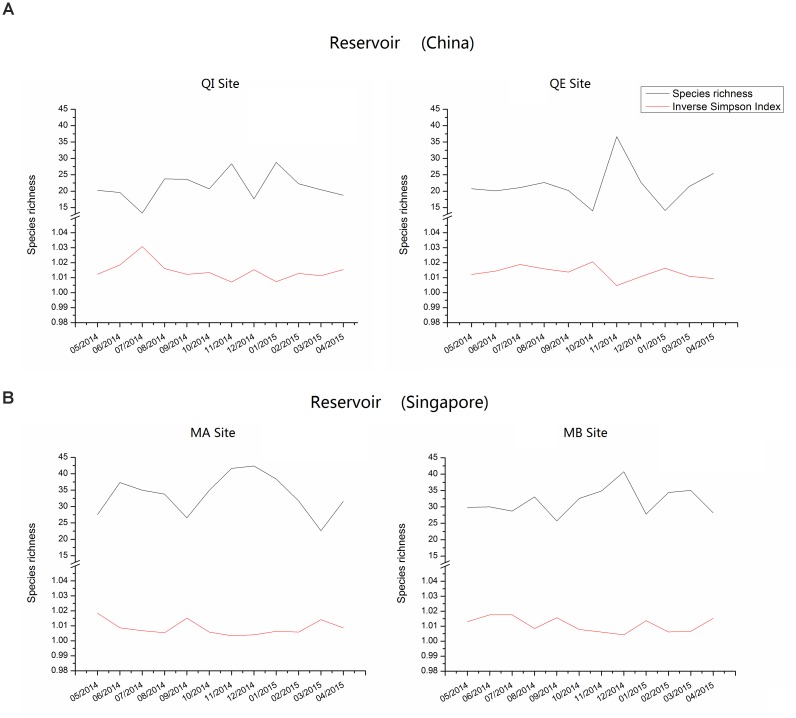
Species Richness and diversity (Inverse Simpson Index) of bacterial community composition in reservoir **A** (China) and **B** (Singapore).

Comparison between two reservoirs showed that the species richness and diversity (inverse Simpson indices) were significant higher in reservoir B than in reservoir A in most of the year (Figure 3). The highest species richness and diversity in reservoir A occurred in November at QE site (Margalet index = 36.63, inverse Simpson index = 1.005), while the lowest values occurred in July at QI site (Margalet index = 13.31, inverse Simpson index = 1.031). In contrast, the highest species richness and diversity in reservoir B occurred in December at MA site (Margalet index = 42.36, inverse Simpson index = 1.004) and the lowest values occurred in March at MA site (Margalet index = 22.62, inverse Simpson index = 1.014).

### Statistical Analysis

Results of ANOSIM and PERMANOVA tests (Supplementary Tables S3, S4) that the bacterial community compositions were significant distinct between different sites in reservoir B (*p* < 0.05), but not in reservoir A. However, temporal differences of bacterial community compositions were found in both reservoirs. In addition, SIMPER test examined the OTUs composition, and found that the spatial and temporal differences of bacterial community composition in each reservoir were mainly attributed to the relative abundance of bacterial OTUs instead of bacterial taxa.

The step-wise DistLM indicated that TN, TN/TP, temperature, chloride, Ca^2+^, Mg^2+^, sulfate, total cyanobacterial 16S gene copies and rainfall (30 d) were the main environmental factors influencing the variations of bacterial community composition in reservoir A (Table 1). In contrast, turbidity, chloride and total cyanobacterial 16S gene copies were dominant factors that significantly affected the variations of bacterial community composition in reservoir B.

**Table 1:** DistLM results of abundant bacterial community data against environmental variables (9,999 permutations).

Variable	Reservoir A				Reservoir B			
	SS(trace)	Pseudo-F	*P*	Prop.	SS(trace)	Pseudo-F	*P*	Prop.
Chl-a	3015.5	1.8793	0.0761	8.21E-02	3083.3	1.4897	0.0546	6.34E-02
pH	2079.7	1.261	0.2228	5.66E-02	2438	1.1615	0.2207	5.01E-02
Turbidity	3186.6	1.9961	0.0642	8.68E-02	4233.6	2.0985	**0.0044**	8.71E-02
TN	11809	9.9578	**0.0001**	0.32166	2973.7	1.4333	0.0689	6.12E-02
TP	2845.6	1.7645	0.0939	7.75E-02	3132	1.5149	0.0515	6.44E-02
TN/TP	6276.7	4.3309	**0.0006**	0.17097	2371.8	1.1283	0.2496	4.88E-02
Temperature	12128	10.36	**0.0001**	0.33035	2601.3	1.2437	0.1566	5.35E-02
Chloride	8729.8	6.5516	**0.0001**	0.23779	3748	1.8377	**0.0121**	7.71E-02
Ca	10699	8.637	**0.0001**	0.29143	1871.2	0.88063	0.6299	3.85E-02
Mg	10304	8.1937	**0.0001**	0.28067	2899.4	1.3952	0.0848	5.96E-02
Sulfate	7216.9	5.1384	**0.0009**	0.19658	2807.4	1.3482	0.1064	5.77E-02
CYAN	3600	2.2832	**0.0229**	9.81E-02	4510.5	2.2498	**0.002**	9.28E-02
Rainfall (30 d)	7893.8	5.7523	**0.0002**	0.21502	2665.6	1.2762	0.1382	5.48E-02

*Bold = Significantly correlated with community structure at α = 0.05.*

**FIGURE 4:**
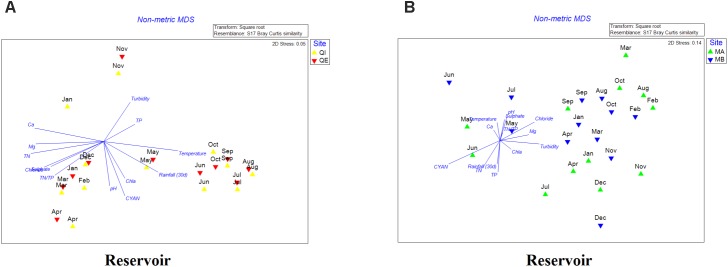
**(A,B)** Non-metric multidimensional scaling (nMDS) plot of the bacterial community dissimilarity (Bray–Curtis) between samples of reservoir A **(A)** and reservoir B **(B)**.

The two-dimensional nMDS plot of reservoir A showed that most of samples presented significant seasonal distribution, where samples from May to October were correlated with high temperature, chlorophyll-a, pH, total cyanobacterial 16S gene copies and rainfall (30 d); whereas samples from November to April were more correlated with high concentrations of chloride, sulfate, Ca^2+^, Mg^2+^, TN and TN/TP (Figure 4A). For reservoir B, the seasonal distinctions were not as significant as in reservoir A, but samples were quite different between dry (June–September) and rain season (December–March). In addition, most samples were correlated with chloride, Mg^2+^, turbidity and chl-a (Figure 4B).

### Multivariate Analyses of Biotic and Abiotic Components

The abundance of bacterial OTUs which contributed >1% to any sample in each reservoir were selected, amounting for a total of 58 bacterial OTUs in reservoir A and 85 bacterial OTUs in reservoir B, respectively. Combined with 15 environmental variables including chl-a, pH, turbidity, TN, TP, TN/TP, temperature, chloride, sulfate, Ca^2+^, Mg^2+^, total cyanobacterial 16S gene copies and rainfall (1 d, 5 d, 30 d) in each reservoir were selected to calculate the linear pairwise correlations using the rcor.test in the ltm package. Finally, 367 tests were considered significant (*p* < 0.05) from a total of 2,628 tested correlations in reservoir A. Among these significant correlations, 243 were positive correlations (including 70 nodes) and 124 were negative (including 50 nodes). In contrast, 335 tests were considered significant (*p* < 0.05) from a total of 4,950 tested correlations in reservoir B (Supplementary Table S5). Within these significant correlations, 325 were positive correlations (including 93 nodes) and 10 were negative (including 16 nodes). In each reservoir, the significant correlations (positive/negative) were further used to construct the co-occurrence/exclusion network based on Fruchterman Reingold algorithm, respectively.

**FIGURE 5:**
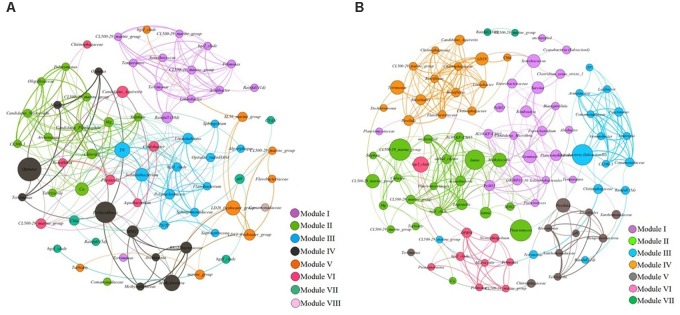
**(A,B)** The network analysis showing the co-occurrence patterns between biotic/abiotic factors and bacterial OTUs in reservoir A **(A)** and reservoir B **(B)**. The nodes were colored according to modularity class. The boldness of line indicates the degree of the correlation. The size of each node is proportional to the values of betweenness centrality.

In co-occurrence pattern (positive correlations) networks of reservoir A, all nodes were clustered into eight modules after modularity classification, including module I (18.57%), module II (18.57%), module III (17.14%), module IV (14.29%), module V (11.43%,), module VI (10%), module VII (8.57%), and module VIII (1.43%) (Figure 5A). In module I, temperature and rainfall (1 d and 30 d) were the most dominant environmental factors, also including 10 bacterial OTUs as *Synechococcus* (Cyanobacteria), *hgcI_clade* (Actinobacteria), *CL500-29_marine_group* (Actinobacteria), *Limnobacter* (β-Proteobacteria), *Acidibacter* (γ-Proteobacteria), *Terrimonas* (Bacteroidetes), and *Filimonas* (Bacteroidetes). In module II, the major important environmental factors were Ca^2+^, Mg^2+^ and SO_42-_, also including nine bacterial OTUs as *CL500-29_marine_group* (Actinobacteria), *Candidatus_Planktophila* (Actinobacteria), *Tabrizicola* (α-Proteobacteria), *Comamonadaceae* (β-Proteobacteria), *Polaromonas* (β-Proteobacteria), *Arenimonas* (γ-Proteobacteria), *Oligoflexaceae* (δ-Proteobacteria), *Candidatus_Nostocoida* (Planctomycetes) and *CL500-3* (Planctomycetes). In Module III, TN/TP was the only environmental factor, and 10 bacterial OTUs including *hgcI_clade* (Actinobacteria), *Sphingomonadaceae* (α-Proteobacteria), *Sphingobium* (α-Proteobacteria), *Limnohabitans* (β-Proteobacteria), *Polynucleobacter* (β-Proteobacteria), *Sediminibacterium* (Bacteroidetes), *Flavobacterium* (Bacteroidetes), *Algoriphagus* (Bacteroidetes), *Saprospiraceae* (Bacteroidetes), *Opitutae_vadinHA64* (Verrucomicrobia) were also within this module. These three modules almost accounted for 54.28% of total nodes in the whole network, which implied that the changes of these environmental variables and bacterial OTUs could strongly affect the whole bacterial community composition. In addition, module IV and VI only composed by different bacterial OTUs. These indicated that bacterial OTUs in module IV and VI were less affected by changes of environmental factors.

In contrast, the co-occurrence pattern network of reservoir B exhibited distinct constitutive characteristics, which further clustered into seven different modules (Figure 5B). Within these modules, Module I and II accounted for 22.58% of the whole network, respectively. Followed by module III (16.13%), module IV (16.13%), module V (10.75%), module VI (9.68%) and module VII (2.15%). In module I, temperature was the dominant environmental factor. And 20 bacterial OTUs exhibited strong co-occurrence correlations with temperature, which including *PeM15* (Actinobacteria), *Candidatus_Microthrix* (Actinobacteria), *Solirubrobacterales* (Actinobacteria), *Synechococcus* (Cyanobacteria), *JG30-KF-CM45* (Chloroflexi), *Sarcina* (Firmicutes), *Peptoclostridium* (Firmicutes), *Clostridium_sensu_stricto_1* (Firmicutes), *Romboutsia* (Firmicutes), *Planctomycetaceae* (Planctomycetes), *Blastopirellula* (Planctomycetes), *Gemmata* (Planctomycetes), *Alsobacter* (α-Proteobacteria), *Enterobacteriaceae* (γ-Proteobacteria), *GR-WP33-30* (δ-Proteobacteria) and so on. In module II, the turbidity, Ca^2+^, Mg^2+^, Cl^-^ and SO_42-_ were the dominant abiotic factors. And co-occurred with bacterial OTUs including *Iamia* (Actinobacteria), *hgcI_clade* (Actinobacteria), *CL500-29_marine_group* (Actinobacteria), *JG30-KF-CM45* (Chloroflexi), *Planctomycetaceae* (Planctomycetes), *Planctomyces* (Planctomycetes), *MNG7* (α-Proteobacteria), *Methylocystis* (α-Proteobacteria), *alphaI_cluster* (α-Proteobacteria) and *Legionella* (γ-Proteobacteria) exhibited significant co-occurrence correlations with these environmental factors. The dominant environmental factors in Module III were TP and rainfall (1 d and 5 d), which co-occurred with bacterial OTUs as *Armatimonas* (Armatimonadetes), *CL500-29_marine_group* (Actinobacteria), *Lacibacter* (Bacteroidetes), *Chitinophagaceae* (Bacteroidetes), *Terrimonas* (Bacteroidetes), *Cyanobacteria_SubsectionIII* (Cyanobacteria), *CL500-3* (Planctomycetes), *Gemmobacter* (α-Proteobacteria), *Comamonadaceae* (β-Proteobacteria), *Paucimonas* (β-Proteobacteria), *Lautropia* (β-Proteobacteria) and *Comamonadaceae* (β-Proteobacteria). But in module IV, the chl-a was the dominant effective factor, co-occurred with bacterial OTUs as *CL500-29_marine_group* (Actinobacteria), *Terrimonas* (Bacteroidetes), *Candidatus_Aquirestis* (Bacteroidetes), *Chitinophagaceae* (Bacteroidetes), *Flavobacteriaceae* (Bacteroidetes), *Roseiflexus* (Chloroflexi), *Roseiflexus* (Chloroflexi), *Pirellula* (Planctomycetes), *LD29* (Verrucomicrobia), *Rhizobiales* (α-Proteobacteria), *Limnobacter* (β-Proteobacteria), and *Dechloromonas* (β-Proteobacteria). These four modules accounted for almost 77.4% nodes of the whole network.

**FIGURE 6:**
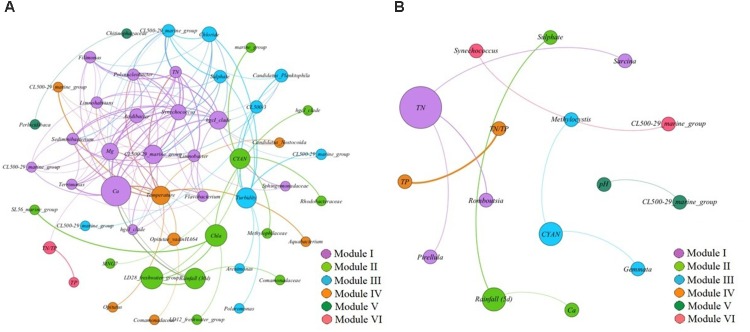
**(A,B)** The network analysis showing the co-exclusion patterns between biotic/abiotic factors and bacterial OTUs in reservoir A **(A)** and reservoir B **(B)**. The nodes were colored according to modularity class. The boldness of line indicates the degree of the correlation. The size of each node is proportional to the values of betweenness centrality.

The co-exclusion pattern networks between two reservoirs revealed differences in composing characteristics (Figures 6A,B). In reservoir A, the whole co-exclusion pattern network was composed by six different modules (Figure 6A). Module I was accounted for 34% of all nodes within the network, followed by module II (24%), module III (20%), module IV (14%), module V (4%) and module VI (4%). In module I, TN, Ca^2+^ and Mg^2+^ were the major environmental variables, and co-excluded with 14 bacterial OTUs including *Synechococcus* (Cyanobacteria), *hgcI_clade* (Actinobacteria), *CL500-29_marine_group* (Actinobacteria), *Terrimonas* (Bacteroidetes), *Sediminibacterium* (Bacteroidetes), *Flavobacterium* (Bacteroidetes), *Filimonas* (Bacteroidetes), *Sphingomonadaceae* (α-Proteobacteria), *Limnohabitans* (β-Proteobacteria), *Limnobacter* (β-Proteobacteria), *Polynucleobacter* (β-Proteobacteria), and *Acidibacter* (γ-Proteobacteria). In module II, the major influencing factors were rainfall (30 d), chl-a and the relative abundance of cyanobacterial copies among the total 16S rRNA in each sample. Also the co-excluded bacterial OTUs were *Holophagaceae* (Acidobacteria), *hgcI_clade* (Actinobacteria), *SL56_marine_group* (Chloroflexi), *MNG7* (α-Proteobacteria), *Rhodobacteraceae* (α-Proteobacteria), *LD12_freshwater_group* (α-Proteobacteria), *Comamonadaceae* (β-Proteobacteria), *LD28_freshwater_group* (β-Proteobacteria) and *Methylophilaceae* (β-Proteobacteria). In module III, turbidity, Cl^-^ and SO_42-_ were the important environmental influencing factors, and the co-excluded bacterial OTUs were *CL500-29_marine_group* (Actinobacteria), *Candidatus_Planktophila* (Actinobacteria), *CL500-3* (Chloroflexi), *Polaromonas* (β-Proteobacteria), and *Arenimonas* (γ-Proteobacteria). These three modules accounted for 78% of nodes in the whole network.

Although the co-excluded pattern network in reservoir B was clustered into six different modules, the composing characteristics of reservoir B was much simple, including less bacterial OTUs and environmental variables (Figure 6B). Module I accounted for 25% of total nodes in the whole network, which composed by TN and co-excluded bacterial OTUs as *Romboutsia* (Firmicutes), *Sarcina* (Firmicutes) and *Pirellula* (Planctomycetes). Module II accounted for 18.75% of total network, and was composed by rainfall (5 d), Ca^2+^ and SO_42-_, which implied that no bacterial OTUs co-excluded with these environmental factors at the same time. Module III accounted for 18.75% of total network, and was composed by the relative abundance of cyanobacterial copies among the total 16S rRNA in each sample, and co-excluded bacterial OTUs as *Gemmata* (Planctomycetes) and *Methylocystis* (α-Proteobacteria). These three modules almost accounted for 62.5% of total network. In addition, *Synechococcus* exhibited co-excluded correlation with *CL500-29_marine_group* (Actinobacteria).

## Discussion

Although many studies have been conducted in order to gain insights into microbial community diversity and dynamic variation in freshwater and estuarine ecosystems of the world, still little is known about the composing characteristics of bacteria in estuary ecosystems between different regions. Here, we provide a comprehensive evaluation by using the Illumina MiSeq sequencing technology and co-occurrence/exclusion pattern network analysis, also examined different influencing environmental factors on correlations of cyanobacteria–heterotrophic bacteria compositions between two reservoirs.

### Differences in Bacterial Community Composition Between the Two Reservoirs

In this study, we found significant differences of bacterial community compositions between two estuarine reservoirs, mainly reflected in taxonomic composition, community diversity and interactions within molecular ecological networks (Figures 5A,B, 6A,B). In comparison, the species richness and diversity of bacterial community composition in reservoir B were relative higher, and exhibited more co-occurrence correlations and less co-exclusion interactions within the molecular ecological networks (Figures 6A,B). These implied that the trophic structure of reservoir B was more suitable for bacterial growth, and reduced bacterial survival pressures on nutrient utilizations. Thus, the bacterial community in reservoir B showed higher diversity and more mutually beneficial co-occurrence correlations. Related study also indicated that the suitable trophic structure could increase the diversity of bacterial community and reduced the competitive pressures between bacteria in aquatic ecosystems (Monier et al., 2015). In reservoir A, the patterns of bacterial community composition were more complexity than in reservoir B, and the bacterial community showed more correlations with the measured environmental variables, which indicated that the variation of bacterial community to the changing environmental conditions was more sensitive. We speculated that the environmental heterogeneity in reservoir A was relative higher than in reservoir B, and finally resulted in expected differences of bacterial community composition between two estuarine reservoirs. The molecular ecological network indicated that the bacterial community showed different responses under different dominant environmental conditions (Ayaz and Gothalwal, 2014; Cornforth et al., 2014). These differences mainly manifested in different modules within the network.

In reservoir A, temperature and rainfall (30 d) were the most important environmental factors in the summer, which further increased the relative abundance of bacterial OTUs such as *Synechococcus* (Cyanobacteria), *CL500-29_marine_group* (Actinobacteria), *hgcI_clade* (Actinobacteria), *Terrimonas* (Bacteroidetes), *Limnobacter* (β-Proteobacteria), and *Acidibacter* (γ-Proteobacteria) (module I). It is well known that the high temperature could promote the growth of Cyanobacteria (Paerl et al., 2001; Paerl and Huisman, 2010), but still not clear how the temperature affected the variations of these non-cyanobacterial relative abundance. Although related study indicated the Actinobacteria have high tolerant to UV transparency (Warnecke et al., 2005), still unknown the UV stress resistance mechanisms of other bacteria, which exhibited higher relative abundance in summer. During other periods, the TN and salt ions (such as Ca^2+^, Mg^2+^ and Cl^-^) were the dominant environmental factors influenced the bacterial community composition (modules II and III). These implied that bacterial OTUs in module II and III have high requirements for TN and salt ions during their metabolic processes, also played important roles in the progresses of N cycle and ion exchanges in aquatic ecosystem. In addition, although the bacterial OTUs as *Opitutus* and *Perlucidibaca* in module IV independent with measured environmental variables, played key roles in the whole network with high betweenness centrality. These implied that *Opitutus* and *Perlucidibaca* have wide connections with other bacteria. Research indicated that the minor bacterial phyla in freshwater lakes might play key roles in the whole bacterial community, but not depend upon their less relative abundance (Lindh et al., 2015).

The molecular ecological network in reservoir B reflected weak interactions between bacterial OTUs and measured environmental variables, which indicated that the reservoir B located in tropical region, the seasonal differences in reservoir B were less noticeable than those in reservoir A. Therefore, the function roles and interactions between bacterial OTUs in the network of reservoir B became more prominent than in reservoir A. Within the co-occurrence pattern network in reservoir B, bacterial OTUs such as *CL500-29_marine_group* (Actinobacteria), *Iamia* (Actinobacteria), *Planctomyces* (Planctomycetes), *LD29* (Verrucomicrobia), and *Synechococcus* (Cyanobacteria) exhibited high betweenness centrality, implied that these bacterial OTUs had wide correlations with other bacterial OTUs and played pivotal roles in co-occurrence network construction of reservoir B. But we still less known about the ecological functions of these bacteria in practical environmental conditions. Thus, further validation is needed under laboratory conditions.

Through the above comparison, we found that the key bacterial compositions (high betweenness centrality) were different between two reservoirs. In reservoir A, dominant heterotrophic bacterial OTUs such as *Filimonas*, *Acidibacter*, hgcI_clade, *Limnobacter*, and *Terrimonas* in the summer, exhibited close correlations with carbon metabolisms. But the functional bacterial taxa including *Sediminibacterium*, *Rhodobacter*, *Limnohabitans*, *Polynucleobacter*, and CL500-29_marine_group, were found increased in relative abundance from winter to spring. Most of these bacterial OTUs exhibited close correlations with TN and salt ions. We speculated that the large number of heterotrophic bacterial taxa multiplied rapidly, which depended on the increased dissolved organic carbon (DOC) loading or alga-derived DOC inputs during summer. But in the winter and spring, the water temperature and rainfall decreased, but the concentrations of TN and salt ions increased significantly. Therefore, the bacterial taxa related with nitrogen and ion metabolisms became dominant. In reservoir B, although the bacterial community exhibited less correlations with environmental factors, these did not mean the dependent extent of bacterial community to environmental also reduced. In addition, bacterial OTUs closely correlated with TP (such as *Armatimonas*, *Lacibacter*, *Paucimonas*, and Comamonadaceae) exhibited higher advantage than in reservoir A, which might correlated with relative higher concentration of TP in reservoir B. In addition, CL500-29_marine_group exhibited co-occurrence correlations with salt ions including Ca^2+^, Mg^2+^, Cl^-^ and SO_42-_ in both two reservoirs. These implied that the CL500-29_marine_group played important roles during the processes of salt ionic conversions in these estuarine reservoirs. Based on the above analysis, we speculated that the ecological functions (such as nutrient and ion metabolisms) of these key bacterial taxa in each reservoir were significant distinct, which mainly depend on the located environmental conditions of reservoirs (Dennis et al., 2013). However, the molecular biological technologies (including HTS technology and metagenomics) still hard to know the specific functional roles of different bacterial taxa under real ecological environmental conditions.

### The Distinctions in Composing Characteristics of Cyanobacteria–Heterotrophic Bacteria Between Two Estuarine Reservoirs

In aquatic ecosystem, Cyanobacteria could release a large number of secondary metabolites during the process of growth, such as DOC and other micro-molecular organics to surroundings. These organic matters could attract some other heterotrophic bacteria, which could decompose and use these metabolites (Yang et al., 2017). On the other hand, these heterotrophic bacteria could release inorganic nutrients during the course of metabolism, which could be recycled or reused by Cyanobacteria (Cai et al., 2014). Thus, a symbiotic and mutually beneficial relationship was built between Cyanobacteria and non-cyanobacteria, which appeared as co-occurrence pattern in bacterial-cyanobacterial systems. Related research has indicated that different cyanobacterial species had specific co-occurred/excluded bacterial community composition within aquatic ecosystems (Louati et al., 2015; Yang et al., 2016). But in our study, same *Synechococcus* also exhibited different composing characteristics of co-occurred/excluded bacterial community between different estuarine reservoirs.

In reservoir A, the dominant *Synechococcus* exhibited co-occurrence correlations with *CL500-29_marine_group* (Actinobacteria), *hgcI_clade* (Actinobacteria), *Terrimonas* (Bacteroidetes), *Limnobacter* (β-Proteobacteria), and *Acidibacter* (γ-Proteobacteria), which both appeared in module I of the co-occurrence pattern network (Figure 5A). In addition, the dominant *Synechococcus* co-excluded with Polynucleobacter (β-Proteobacteria), *Limnohabitans* (β-Proteobacteria), *Sediminibacterium* (Bacteroidetes), *Flavobacterium* (Bacteroidetes) and *Opitutae_vadinHA64* (Verrucomicrobia) in co-exclusion pattern network (Figure 6A). These results were consistent with previous conclusions that some kinds of β-Proteobacteria and Bacteroidetes could promote or inhibit the growth of the coexisting Cyanobacteria (Lemarchand et al., 2006; Eiler and Bertilsson, 2007; Eiler et al., 2010; Zeder et al., 2010; Newton et al., 2011). We speculate that potential interactions between these bacterial taxa and *Synechococcus* might exist. For instance, a spatial niche competition or nutrient mutualism may happen. However, besides the actinobacterial taxa were not found, apparently it does not have any physical association with Cyanobacteria in previous study (Kolmonen et al., 2004; Allgaier et al., 2007). These implied that might exist other unknown interactions between these actinobacterial taxa and *Synechococcus* (Salcher et al., 2010). In addition, the concentrations of TN and salt ions (Ca^2+^, Mg^2+^, Cl^-^ and SO_42-_) significantly inhibited the increased *Synechococcus*, which indicated that the variation of *Synechococcus* was affected by multi-environmental factors in reservoir A.

Although the water environmental characteristics were much different between two estuarine reservoirs, the *Synechococcus* became the most dominant cyanobacterial species in both reservoirs. It is unclear why *Synechococcus* became dominant in these reservoirs. In addition, the network indicated that the co-occurred bacterial OTUs were much different in reservoir B, which composed by *Clostridium_sensu_stricto_1* (Firmicutes), *Sarcina* (Firmicutes) and *LD29* (Verrucomicrobia). It is noted that the *LD29* as key node within the network, widely correlated with other bacterial OTUs (Figure 5B). Although these bacterial OTUs were not cosmopolitan bacterial phyla in aquatic ecosystems such as Actinobacteria and Proteobacteria, still had important roles and closely affected the composition of bacterioplankton community. While, due to technical restriction, the ecological functions of these bacterial OTUs are still not clear. In addition, the *CL500-29_marine_group* (Actinobacteria) exhibited co-exclusion pattern with increased *Synechococcus* in reservoir B (Figure 6B). This was contradictory to what was found in reservoir A, which indicated that the relationships of cyanobacteria and heterotrophic bacteria population was dynamic, and fluctuating.

Besides, with the exception of *Synechococcus*, *Microcystis*, and *Pseudanabeana* were also found with relative higher abundance in reservoir B. While the *Microcystis* and *Pseudanabeana* exhibited closed co-occurrence patterns, and both co-occurred with *hgcI_clade* (Actinobacteria), OPB56 (Chlorobi), Filimonas (Bacteroidetes) and Novosphingobium (α-Proteobacteria) (Figure 5B). Compared with the *Microcystis*, the *Pseudanabeana* had ability of nitrogen fixation function in aquatic ecosystems. Combined with previous research results, we speculated that the non-N-fixing *Microcystis* accompanied with N-fixing *Pseudanabeana* occurrence in freshwater lakes, so as to better meet the demand for nitrogen source (Paerl and Otten, 2016).

## Conclusion

This study highlights the comparison of microbial community, especially the characteristics and dynamics of cyanobacteria–heterotrophic bacteria in two estuarine reservoirs between tropical and sub-tropical regions. The bacterial community compositions were significantly different between two reservoirs, and mainly affected by different local environmental factors. The environmental heterogeneity in reservoir A was much higher, which indicated that the composition of bacterial community in reservoir A was more complex. The temperature, environmental conditions and nutritional status were suitable in reservoir B. Thus, the bacterial community in reservoir B have high diversity and less co-exclusion correlations. Although the *Synechococcus* was the dominant Cyanobacteria in both reservoirs, the composing characteristics of cyanobacteria–heterotrophic bacteria were significant distinct between two reservoirs. Also, the correlations between bacteria and cyanobacteria exhibited highly dynamic variations, which was affected by nutrition and survive space. In addition, the *Microcystis* and *Pseudanabeana* in reservoir B exhibited co-occurring patterns, which implied in potential association due to nutrient sources utilization.

## Author Contributions

YH designed this research, and in charged of experiment in China. ZX performed experiments, analyzed data, prepared the figures, and wrote the manuscript text. ST helped sampling in Singapore and data analysis. KG in charged of experiment in Singapore and revised our manuscript earnestly. All authors have contributed and approved the final manuscript.

## Conflict of Interest Statement

The authors declare that the research was conducted in the absence of any commercial or financial relationships that could be construed as a potential conflict of interest.

## Footnotes

1 http://www.mothur.org/

2 http://www.arb-silva.de

3 https://gephi.org/
