# Supplementary material for: The Characteristics and Dynamics of Cyanobacteria–Heterotrophic Bacteria Between Two Estuarine Reservoirs – Tropical Versus Sub-Tropical Regions
Source: Front Microbiol. 2018 Nov 6;9:2531. doi: 10.3389/fmicb.2018.02531 (PMC6232297; doi:10.3389/fmicb.2018.02531)
Supplement: Supplementary file 1 [file Data_Sheet_1.docx]

**Supplementary Material**

[**Frontiers**](http://www.letpub.com.cn/index.php?page=journalapp&view=detail&journalid=2819) **in Microbiology**

**Characteristics comparison of bacterial community composition between two estuarine reservoirs from a tropical and sub-tropical region**

Zheng Xu ^a^, Shu Harn Te ^b­^, Yiliang He ^a^^,^ *, Karina Yew-Hoong Gin ^b, c,^ *

^a^ *School of Environmental Science and Engineering, Shanghai Jiao Tong University, Shanghai 200240, PR China*

^b^ *NUS Environmental Research Institute (NERI), National University of Singapore, Singapore 138602*

^c^ *Department of Civil and Environmental Engineering**, National University of Singapore, Singapore 138602*

** Corresponding author. Tel.: +86 21 54744008; E-mail address: ylhe@sjtu.edu.cn*

**Supplementary Material**

**
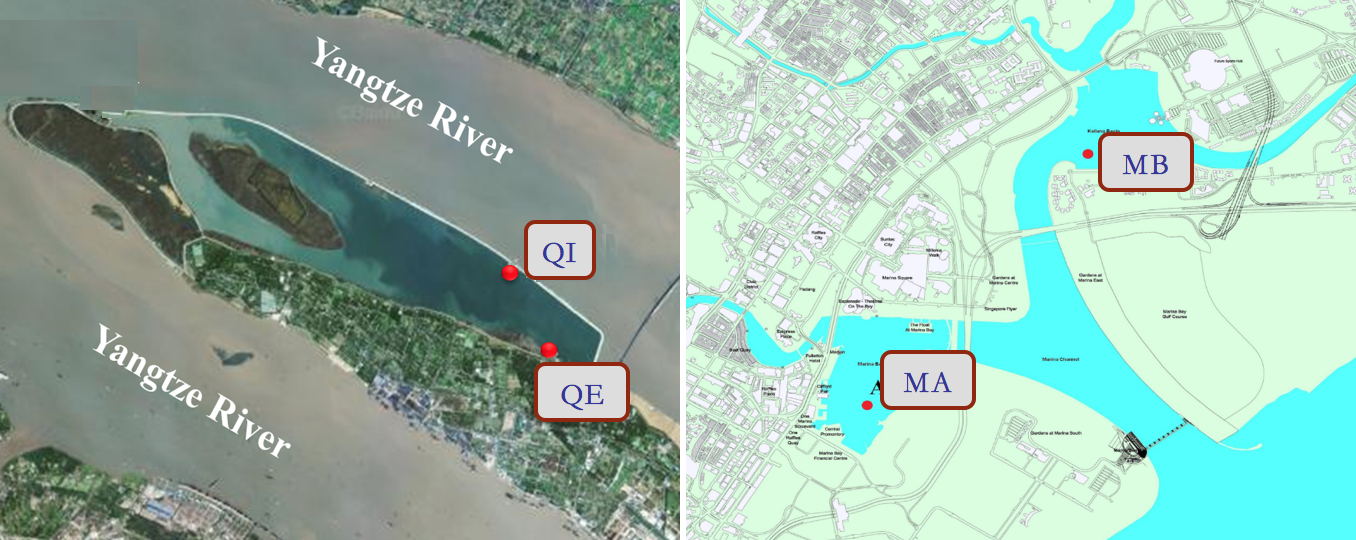
**

Fig. S1 Aerial schematic of two different estuarine reservoirs and annotated sampling

(reservoir A: Qingcaosha Reservoir in Shanghai, China (left); reservoir B: Marina Reservoir in Singapore (right))


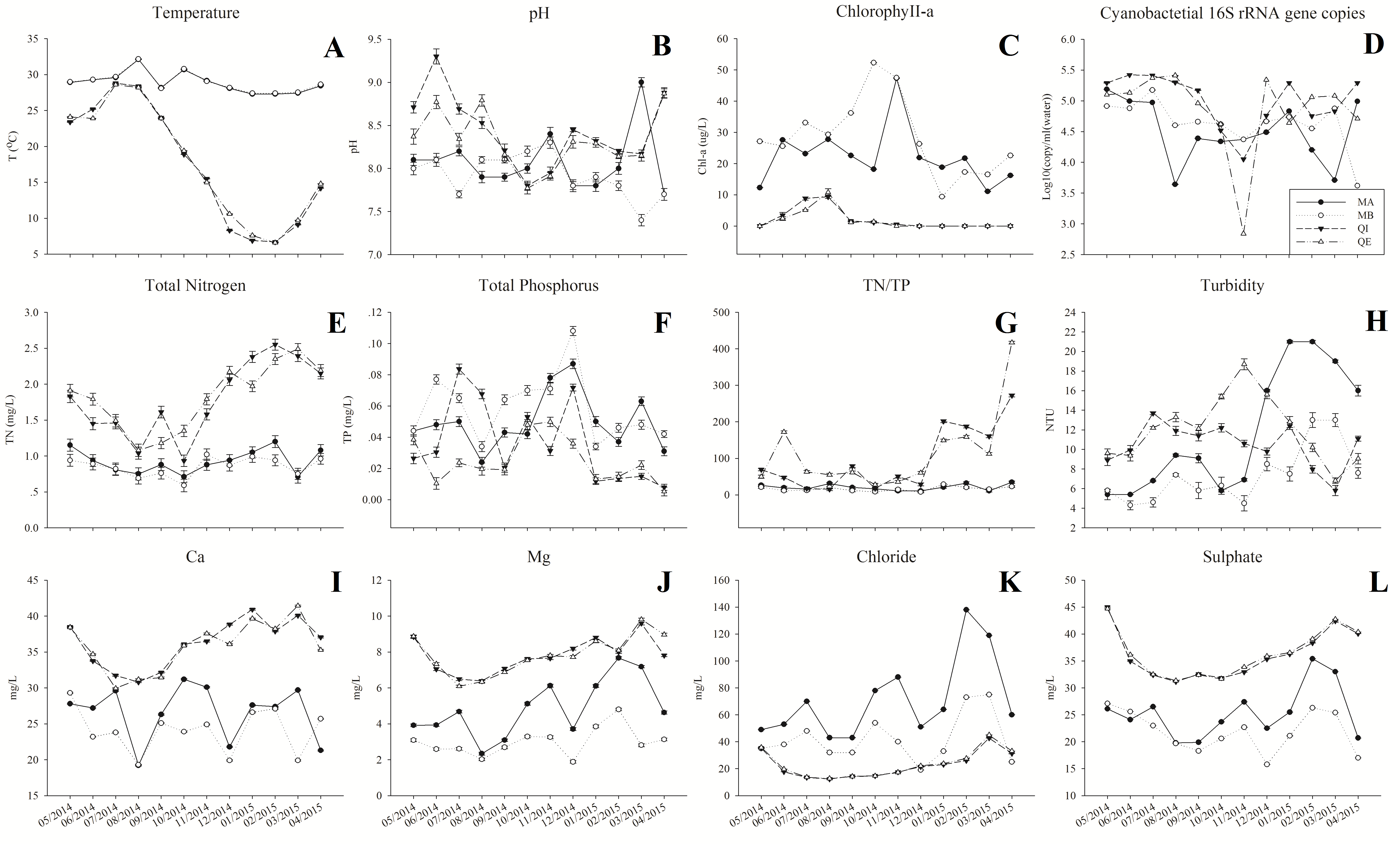


Fig. S2 Water chemistry and environmental parameters in reservoir A and B (A: Temperature, B: pH, C: Chlorophyll-a, D: Cyanobacterial 16S rRNA gene copies, E: Total Nitrogen, F: Total Phosphorus, G: TN/TP, H: Turbidity, I: Ca, J: Mg, K: Chloride, L: Sulphate)

Table S1 Supplemental water chemistry and environmental parameters in Reservoir A

| reservoir A | Rainfall (1d) | Rainfall (5d) | Rainfall (30d) |
| --- | --- | --- | --- |
| QI_2014_05 | 0 | 1.9 | 61.5 |
| QI_2014_06 | 0 | 33.2 | 175.9 |
| QI_2014_07 | 0 | 0 | 192.2 |
| QI_2014_08 | 0.01 | 43.7 | 229.3 |
| QI_2014_09 | 3.68 | 42.9 | 196 |
| QI_2014_10 | 0 | 0 | 37.3 |
| QI_2014_11 | 0.15 | 11.7 | 34.6 |
| QI_2014_12 | 0 | 0 | 5.8 |
| QI_2015_01 | 0.46 | 4.6 | 61.1 |
| QI_2015_02 | 0 | 1.2 | 81.1 |
| QI_2015_03 | 0.08 | 31.2 | 96.4 |
| QI_2015_04 | 0 | 59.5 | 108.9 |
| QE_2014_05 | 0 | 1.9 | 61.5 |
| QE_2014_06 | 0 | 33.2 | 175.9 |
| QE_2014_07 | 0 | 0 | 192.2 |
| QE_2014_08 | 0.01 | 43.7 | 229.3 |
| QE_2014_09 | 3.68 | 42.9 | 196 |
| QE_2014_10 | 0 | 0 | 37.3 |
| QE_2014_11 | 0.15 | 11.7 | 34.6 |
| QE_2014_12 | 0 | 0 | 5.8 |
| QE_2015_01 | 0.46 | 4.6 | 61.1 |
| QE_2015_03 | 0.08 | 31.2 | 96.4 |
| QE_2015_04 | 0 | 59.5 | 108.9 |

Table S2 Supplemental water chemistry and environmental parameters in Reservoir B

| reservoir B | Rain (1d) | Rain (5d) | Rain (30d) |
| --- | --- | --- | --- |
| MA_2014_05 | 0 | 2 | 242.7 |
| MA_2014_06 | 10.8 | 16.4 | 100.5 |
| MA_2014_07 | 8.8 | 14.6 | 62.7 |
| MA_2014_08 | 11.8 | 46.8 | 209.79 |
| MA_2014_09 | 0.2 | 32.6 | 155.7 |
| MA_2014_10 | 0 | 0 | 111.6 |
| MA_2014_11 | 6.8 | 6.8 | 104.1 |
| MA_2014_12 | 0.6 | 58.8 | 282 |
| MA_2015_01 | 0 | 0.8 | 400.5 |
| MA_2015_02 | 0 | 0.2 | 83.7 |
| MA_2015_03 | 0 | 0 | 49.5 |
| MA_2015_04 | 32.79 | 78.4 | 135.9 |
| MB_2014_05 | 0 | 2 | 242.7 |
| MB_2014_06 | 10.8 | 16.4 | 100.5 |
| MB_2014_07 | 8.8 | 14.6 | 62.7 |
| MB_2014_08 | 11.8 | 46.8 | 209.79 |
| MB_2014_09 | 0.2 | 32.6 | 155.7 |
| MB_2014_10 | 0 | 0 | 111.6 |
| MB_2014_11 | 6.8 | 6.8 | 104.1 |
| MB_2014_12 | 0.6 | 58.8 | 282 |
| MB_2015_01 | 0 | 0.8 | 400.5 |
| MB_2015_02 | 0 | 0.2 | 83.7 |
| MB_2015_03 | 0 | 0 | 49.5 |
| MB_2015_04 | 32.79 | 78.4 | 135.9 |

Table S3. PERMANOVA analysis of the Bray-Curtis dissimilarities for bacterial community composition in reservoir A. Df = degrees of freedom; SS = sum of squares; MS = mean sum of squares; Pseudo-F = F value by permutation, P(perm) = P-values based on 9999 permutations.

| Bacterial community Source | df | SS | MS | Pseudo-F | P(perm) |
| --- | --- | --- | --- | --- | --- |
| Site | 1 | 657.32 | 657.32 | 1.1147 | 0.3775 |
| Time | 11 | 30085 | 2735 | 4.6382 | 0.0001 |
| Res | 10 | 5896.7 | 589.67 |  |  |
| Total | 22 | 36712 |  |  |  |

Table S4. PERMANOVA analysis of the Bray-Curtis dissimilarities for bacterial community composition in reservoir B. Df = degrees of freedom; SS = sum of squares; MS = mean sum of squares; Pseudo-F = F value by permutation, P(perm) = P-values based on 9999 permutations.

| Bacterial community Source | df | SS | MS | Pseudo-F | P(perm) |
| --- | --- | --- | --- | --- | --- |
| Site | 1 | 2970.5 | 2970.5 | 2.2211 | 0.0057 |
| Time | 11 | 30935 | 2812.2 | 2.1027 | 0.0001 |
| Res | 11 | 14712 | 1337.4 |  |  |
| Total | 23 | 48617 |  |  |  |

Table S5. Multiple linear correlations by rcor.test in ltm package (R language).

| A | B | R |
| --- | --- | --- |
| Otu000012 | Otu000003 | 0.72 |
| Otu000018 | Otu000017 | 0.88 |
| Otu000022 | Otu000012 | 0.73 |
| Otu000030 | Otu000012 | 0.73 |
| Otu000030 | Otu000022 | 0.92 |
| Otu000031 | Otu000005 | 0.91 |
| Otu000035 | Otu000003 | 0.73 |
| Otu000038 | Otu000035 | 0.81 |
| Otu000044 | Otu000036 | 0.72 |
| Otu000046 | Otu000017 | 0.77 |
| Otu000048 | Otu000036 | 0.84 |
| Otu000050 | Otu000036 | 0.83 |
| Otu000050 | Otu000044 | 0.78 |
| Otu000050 | Otu000048 | 0.81 |
| Otu000052 | Otu000029 | 0.87 |
| Otu000053 | Otu000005 | 0.74 |
| Otu000058 | Otu000015 | 0.84 |
| Otu000058 | Otu000036 | 0.72 |
| Otu000059 | Otu000029 | 0.88 |
| Otu000059 | Otu000052 | 0.86 |
| Otu000062 | Otu000036 | 0.86 |
| Otu000062 | Otu000040 | 0.77 |
| Otu000062 | Otu000044 | 0.86 |
| Otu000062 | Otu000048 | 0.8 |
| Otu000062 | Otu000050 | 0.78 |
| Otu000064 | Otu000038 | 0.75 |
| Otu000066 | Otu000017 | 0.91 |
| Otu000066 | Otu000018 | 0.88 |
| Otu000067 | Otu000036 | 0.85 |
| Otu000067 | Otu000044 | 0.76 |
| Otu000067 | Otu000048 | 0.78 |
| Otu000067 | Otu000050 | 0.74 |
| Otu000067 | Otu000062 | 0.88 |
| Otu000070 | Otu000022 | 0.81 |
| Otu000070 | Otu000030 | 0.77 |
| Otu000073 | Otu000007 | 0.71 |
| Otu000073 | Otu000015 | 0.85 |
| Otu000073 | Otu000058 | 0.86 |
| Otu000074 | Otu000007 | 0.74 |
| Otu000074 | Otu000015 | 0.84 |
| Otu000074 | Otu000032 | 0.78 |
| Otu000074 | Otu000058 | 0.85 |
| Otu000074 | Otu000073 | 0.83 |
| Otu000076 | Otu000007 | 0.75 |
| Otu000076 | Otu000015 | 0.78 |
| Otu000076 | Otu000058 | 0.86 |
| Otu000076 | Otu000073 | 0.92 |
| Otu000076 | Otu000074 | 0.82 |
| Otu000077 | Otu000052 | 0.82 |
| Otu000079 | Otu000029 | 0.93 |
| Otu000079 | Otu000052 | 0.75 |
| Otu000079 | Otu000059 | 0.75 |
| Otu000081 | Otu000015 | 0.82 |
| Otu000081 | Otu000036 | 0.77 |
| Otu000081 | Otu000058 | 0.96 |
| Otu000081 | Otu000073 | 0.86 |
| Otu000081 | Otu000074 | 0.8 |
| Otu000081 | Otu000076 | 0.86 |
| Otu000085 | Otu000007 | 0.77 |
| Otu000096 | Otu000029 | 0.79 |
| Otu000096 | Otu000052 | 0.74 |
| Otu000096 | Otu000059 | 0.74 |
| Otu000096 | Otu000077 | 0.89 |
| Otu000096 | Otu000079 | 0.72 |
| Otu000102 | Otu000006 | 0.72 |
| Otu000102 | Otu000064 | 0.74 |
| Otu000102 | Otu000080 | 0.75 |
| Otu000105 | Otu000007 | 0.75 |
| Otu000105 | Otu000015 | 0.79 |
| Otu000105 | Otu000036 | 0.82 |
| Otu000105 | Otu000058 | 0.88 |
| Otu000105 | Otu000073 | 0.8 |
| Otu000105 | Otu000074 | 0.76 |
| Otu000105 | Otu000076 | 0.83 |
| Otu000105 | Otu000081 | 0.93 |
| Otu000106 | Otu000012 | 0.74 |
| Otu000106 | Otu000022 | 0.82 |
| Otu000106 | Otu000030 | 0.79 |
| Otu000106 | Otu000070 | 0.85 |
| Otu000107 | Otu000018 | 0.77 |
| Otu000107 | Otu000040 | 0.73 |
| Otu000107 | Otu000044 | 0.71 |
| Otu000107 | Otu000062 | 0.77 |
| Otu000107 | Otu000075 | 0.75 |
| Otu000107 | Otu000085 | 0.88 |
| Otu000108 | Otu000038 | 0.84 |
| Otu000108 | Otu000047 | 0.77 |
| Otu000108 | Otu000064 | 0.81 |
| Otu000108 | Otu000080 | 0.79 |
| Otu000108 | Otu000102 | 0.78 |
| Otu000109 | Otu000068 | 0.79 |
| Otu000113 | Otu000014 | 0.76 |
| Otu000113 | Otu000047 | 0.74 |
| Otu000113 | Otu000080 | 0.77 |
| Otu000113 | Otu000108 | 0.75 |
| Otu000117 | Otu000005 | 0.95 |
| Otu000117 | Otu000031 | 0.89 |
| Otu000120 | Otu000029 | 0.78 |
| Otu000120 | Otu000052 | 0.89 |
| Otu000120 | Otu000059 | 0.79 |
| Otu000120 | Otu000077 | 0.84 |
| Otu000120 | Otu000096 | 0.75 |
| Otu000121 | Otu000022 | 0.77 |
| Otu000121 | Otu000030 | 0.77 |
| Otu000121 | Otu000070 | 0.74 |
| Otu000126 | Otu000022 | 0.83 |
| Otu000126 | Otu000030 | 0.92 |
| Otu000126 | Otu000070 | 0.75 |
| Otu000137 | Otu000029 | 0.8 |
| Otu000137 | Otu000052 | 0.91 |
| Otu000137 | Otu000059 | 0.81 |
| Otu000137 | Otu000077 | 0.8 |
| Otu000137 | Otu000079 | 0.75 |
| Otu000137 | Otu000096 | 0.76 |
| Otu000137 | Otu000120 | 0.93 |
| Otu000138 | Otu000029 | 0.86 |
| Otu000138 | Otu000052 | 0.96 |
| Otu000138 | Otu000059 | 0.86 |
| Otu000138 | Otu000077 | 0.81 |
| Otu000138 | Otu000079 | 0.75 |
| Otu000138 | Otu000096 | 0.76 |
| Otu000138 | Otu000120 | 0.87 |
| Otu000138 | Otu000137 | 0.93 |
| Otu000146 | Otu000005 | 0.9 |
| Otu000146 | Otu000031 | 0.88 |
| Otu000146 | Otu000117 | 0.88 |
| Otu000147 | Otu000015 | 0.75 |
| Otu000147 | Otu000058 | 0.88 |
| Otu000147 | Otu000073 | 0.78 |
| Otu000147 | Otu000076 | 0.81 |
| Otu000147 | Otu000081 | 0.9 |
| Otu000147 | Otu000105 | 0.83 |
| Otu000150 | Otu000015 | 0.74 |
| Otu000150 | Otu000058 | 0.83 |
| Otu000150 | Otu000073 | 0.74 |
| Otu000150 | Otu000076 | 0.79 |
| Otu000150 | Otu000081 | 0.87 |
| Otu000150 | Otu000105 | 0.81 |
| Otu000150 | Otu000147 | 0.98 |
| Otu000151 | Otu000005 | 0.93 |
| Otu000151 | Otu000031 | 0.9 |
| Otu000151 | Otu000117 | 0.92 |
| Otu000151 | Otu000146 | 0.92 |
| Otu000154 | Otu000102 | 0.72 |
| Otu000154 | Otu000108 | 0.74 |
| Otu000154 | Otu000113 | 0.78 |
| Otu000163 | Otu000030 | 0.73 |
| Otu000163 | Otu000126 | 0.76 |
| Otu000195 | Otu000038 | 0.72 |
| Otu000195 | Otu000047 | 0.78 |
| Otu000195 | Otu000080 | 0.82 |
| Otu000195 | Otu000102 | 0.8 |
| Otu000195 | Otu000108 | 0.93 |
| Otu000195 | Otu000113 | 0.85 |
| Otu000195 | Otu000154 | 0.84 |
| Otu000213 | Otu000047 | 0.76 |
| Otu000213 | Otu000080 | 0.79 |
| Otu000213 | Otu000102 | 0.72 |
| Otu000213 | Otu000108 | 0.88 |
| Otu000213 | Otu000113 | 0.83 |
| Otu000213 | Otu000154 | 0.83 |
| Otu000213 | Otu000195 | 0.99 |
| Otu000219 | Otu000038 | 0.77 |
| Otu000219 | Otu000047 | 0.8 |
| Otu000219 | Otu000064 | 0.72 |
| Otu000219 | Otu000080 | 0.84 |
| Otu000219 | Otu000102 | 0.77 |
| Otu000219 | Otu000108 | 0.95 |
| Otu000219 | Otu000113 | 0.84 |
| Otu000219 | Otu000154 | 0.79 |
| Otu000219 | Otu000195 | 0.97 |
| Otu000219 | Otu000213 | 0.94 |
| Chla | Otu000006 | 0.76 |
| Chla | Otu000102 | 0.81 |
| pH | Otu000151 | 0.75 |
| TN.TP | TP | -0.84 |
| Chloride | Otu000014 | 0.72 |
| Mg | Chloride | 0.88 |
| Sulphate | Chloride | 0.85 |
| Sulphate | Mg | 0.78 |
| Otu000007 | Otu000004 | 0.7 |
| Otu000015 | Otu000014 | 0.7 |
| Otu000016 | Otu000008 | 0.71 |
| Otu000036 | Otu000007 | 0.71 |
| Otu000077 | Otu000029 | 0.71 |
| Otu000080 | Otu000038 | 0.7 |
| Otu000081 | Otu000007 | 0.7 |
| Otu000085 | Otu000075 | 0.7 |
| Otu000097 | Otu000004 | 0.71 |
| Otu000113 | Otu000102 | 0.71 |
| Otu000121 | Otu000012 | 0.7 |
| Otu000154 | Otu000080 | 0.7 |
| Otu000195 | Otu000035 | 0.7 |
| TP | Otu000096 | 0.7 |
| Chloride | Otu000150 | 0.7 |
| Rain..5d. | Otu000079 | 0.7 |
| Otu000022 | Otu000003 | 0.69 |
| Otu000034 | Otu000014 | 0.68 |
| Otu000038 | Otu000008 | 0.69 |
| Otu000047 | Otu000038 | 0.69 |
| Otu000068 | Otu000056 | 0.7 |
| Otu000080 | Otu000047 | 0.68 |
| Otu000107 | Otu000048 | 0.68 |
| Otu000108 | Otu000008 | 0.69 |
| Otu000120 | Otu000079 | 0.68 |
| Otu000121 | Otu000003 | 0.69 |
| Otu000213 | Otu000038 | 0.68 |
| Otu000219 | Otu000035 | 0.69 |
| Temperature | Otu000018 | 0.69 |
| Chloride | Otu000105 | 0.69 |
| Rain..5d. | Rain..1d. | 0.7 |
| Otu000008 | Otu000003 | 0.67 |
| Otu000032 | Otu000007 | 0.67 |
| Otu000044 | Otu000040 | 0.68 |
| Otu000046 | Otu000006 | 0.67 |
| Otu000048 | Otu000044 | 0.67 |
| Otu000055 | Otu000044 | 0.68 |
| Otu000058 | Otu000007 | 0.67 |
| Otu000064 | Otu000047 | 0.67 |
| Otu000073 | Otu000032 | 0.68 |
| Otu000075 | Otu000019 | 0.68 |
| Otu000077 | Otu000059 | 0.67 |
| Otu000104 | Otu000064 | 0.68 |
| Otu000105 | Otu000067 | 0.67 |
| Otu000108 | Otu000035 | 0.68 |
| Otu000121 | Otu000106 | 0.67 |
| Otu000126 | Otu000106 | 0.68 |
| Otu000146 | Otu000053 | 0.67 |
| Otu000147 | Otu000074 | 0.68 |
| Otu000156 | Otu000020 | 0.68 |
| Otu000213 | Otu000035 | 0.68 |
| pH | Otu000146 | 0.67 |
| TN | Otu000066 | -0.67 |
| Otu000044 | Otu000018 | 0.67 |
| Otu000053 | Otu000031 | 0.67 |
| Otu000105 | Otu000014 | 0.66 |
| Otu000105 | Otu000048 | 0.66 |
| Otu000117 | Otu000053 | 0.67 |
| Otu000126 | Otu000121 | 0.66 |
| Chla | Otu000064 | 0.67 |
| Mg | Ca | 0.66 |
| Otu000017 | Otu000006 | 0.66 |
| Otu000034 | Otu000015 | 0.66 |
| Otu000066 | Otu000046 | 0.66 |
| Otu000195 | Otu000064 | 0.66 |
| pH | Otu000005 | 0.66 |
| TP | Otu000077 | 0.66 |
| Sulphate | Otu000014 | 0.66 |
| Rain..5d. | Sulphate | -0.66 |
| Otu000151 | Otu000053 | 0.65 |
| pH | Otu000031 | 0.65 |
| Otu000050 | Otu000018 | 0.64 |
| Chloride | Otu000081 | 0.64 |
| Mg | Turbidity | 0.64 |
| Otu000032 | Otu000004 | 0.64 |
| Otu000037 | Otu000019 | 0.64 |
| Otu000085 | Otu000018 | 0.64 |
| Otu000150 | Otu000074 | 0.64 |
| Otu000154 | Otu000047 | 0.64 |
| Otu000050 | Otu000007 | 0.63 |
| Otu000066 | Otu000040 | 0.63 |
| Otu000080 | Otu000064 | 0.63 |
| Otu000107 | Otu000007 | 0.63 |
| Otu000156 | Otu000053 | 0.63 |
| TP | Otu000120 | 0.63 |
| Otu000047 | Otu000035 | 0.63 |
| Otu000053 | Otu000040 | 0.63 |
| Otu000058 | Otu000014 | 0.63 |
| Otu000076 | Otu000032 | 0.63 |
| Otu000079 | Otu000026 | 0.63 |
| Otu000095 | Otu000031 | 0.63 |
| Otu000102 | Otu000047 | 0.63 |
| Otu000109 | Otu000029 | 0.63 |
| Otu000154 | Otu000014 | 0.63 |
| Otu000016 | Otu000014 | 0.63 |
| Otu000067 | Otu000040 | 0.63 |
| Otu000070 | Otu000012 | 0.63 |
| Otu000097 | Otu000046 | 0.63 |
| Otu000107 | Otu000067 | 0.63 |
| pH | Otu000117 | 0.63 |
| TN | Otu000017 | -0.63 |
| Chloride | Otu000015 | 0.63 |
| Chloride | Otu000147 | 0.62 |
| Chloride | Turbidity | 0.62 |
| Otu000076 | Otu000036 | 0.62 |
| Otu000163 | Otu000022 | 0.62 |
| TN | Otu000053 | -0.62 |
| Otu000032 | Otu000026 | 0.62 |
| Otu000062 | Otu000018 | 0.62 |
| Otu000073 | Otu000036 | 0.62 |
| Otu000107 | Otu000066 | 0.62 |
| Otu000150 | Otu000007 | 0.62 |
| Rain..30d. | Otu000042 | 0.62 |
| Sulphate | Otu000150 | 0.62 |
| Otu000048 | Otu000007 | 0.61 |
| Otu000074 | Otu000023 | 0.61 |
| Otu000075 | Otu000018 | 0.61 |
| Otu000040 | Otu000018 | 0.61 |
| Otu000073 | Otu000014 | 0.61 |
| Sulphate | Otu000015 | 0.61 |
| Otu000014 | Otu000001 | -0.6 |
| Otu000050 | Otu000017 | 0.6 |
| Otu000147 | Otu000007 | 0.6 |
| Temperature | Otu000066 | 0.6 |
| Otu000079 | Otu000077 | 0.6 |
| Otu000074 | Otu000026 | 0.6 |
| Otu000107 | Otu000036 | 0.6 |
| TP | Otu000137 | 0.6 |
| Otu000035 | Otu000008 | 0.59 |
| Otu000107 | Otu000050 | 0.59 |
| Rain..5d. | Otu000029 | 0.59 |
| Otu000074 | Otu000036 | 0.59 |
| Otu000066 | Otu000053 | 0.59 |
| Otu000074 | Otu000004 | 0.59 |
| Otu000213 | Otu000064 | 0.59 |
| Otu000023 | Otu000017 | 0.59 |
| Otu000107 | Otu000095 | 0.59 |
| Temperature | Otu000017 | 0.59 |
| Mg | Otu000014 | 0.59 |
| Otu000044 | Otu000007 | 0.58 |
| Chloride | Otu000058 | 0.58 |
| Otu000023 | Otu000018 | 0.58 |
| Otu000081 | Otu000014 | 0.58 |
| Otu000085 | Otu000044 | 0.58 |
| Otu000109 | Otu000059 | 0.58 |
| Otu000126 | Otu000012 | 0.58 |
| Turbidity | Otu000032 | 0.58 |
| CYAN | Otu000107 | -0.58 |
| pH | Otu000056 | -0.58 |
| CYAN | Otu000007 | -0.58 |
| Rain..5d. | Ca | -0.58 |
| Otu000076 | Otu000014 | 0.58 |
| Otu000034 | Otu000016 | 0.58 |
| Mg | Otu000150 | 0.58 |
| Otu000081 | Otu000067 | 0.58 |
